# Delegates to the State Society

**Published:** 1850-02

**Authors:** 


					﻿At a Quarterly Meeting of the Philadelphia County Medical
Society, the following were elected Delegates to the State Society :
Washington L. Atlee, Henry Bond, John Bell, Governeur Emerson,
George Fox, Henry Gibbons, Hugh L. Hodge, Isaac Hays, Edward
Hallowell, Professor S. Jackson, Samuel Jackson, Joseph Klapp,
J. Forsyth Meigs, George W. Norris, John Neill, Isaac Parrish, Wm.
Byrd Page, H. S. Patterson, Wm. Pepper, M. M. Reeve, Alfred Stille,
George B. Wood, F. West, Benjamin S. Janney, Wilson Jewell, John
D. Logan, Wm. Mayburry, Isaac Remington, Joseph Warrington,
D. Francis Condie, T. H. Yardley, A. Helfenstein, T. F. Betton,
John M. Pugh.
And the following were elected Delegates to the American Medical
Association :
Washington L. Atlee, Henry Bond, John Bell, D. Francis Condie,
Joseph Carson, Governeur Emerson, Wm. R. Grant, Prof. S. Jackson,
S. Jackson, Joseph Klapp, J. H. B. McClellan, G. W. Norris, Isaac
Parrish, Wm. Pepper, Alfred Stille, Francis West, I. Curran.
				

